# Electrochemical Biosensor for Nitrite Based on Polyacrylic-Graphene Composite Film with Covalently Immobilized Hemoglobin

**DOI:** 10.3390/s18051343

**Published:** 2018-04-26

**Authors:** Raja Zaidatul Akhmar Raja Jamaluddin, Lee Yook Heng, Ling Ling Tan, Kwok Feng Chong

**Affiliations:** 1 School of Chemical Sciences and Food Technology, Faculty of Science and Technology, University Kebangsaan Malaysia (UKM), Bangi 43600, Selangor D. E., Malaysia; anne2282@yahoo.com; 2 Southeast Asia Disaster Prevention Research Initiative (SEADPRI-UKM), Institute for Environment and Development (LESTARI), Universiti Kebangsaan Malaysia (UKM), Bangi 43600, Selangor D. E., Malaysia; babybabeoo@gmail.com; 3 Faculty of Industrial Sciences & Technology, Universiti Malaysia Pahang, Lebuhraya Tun Razak, Gambang, Kuantan 26300, Pahang Darul Makmur, Malaysia; ckfeng@ump.edu.my

**Keywords:** edible bird’s nest, electrochemical biosensor, graphene, hemoglobin, nitrite

## Abstract

A new biosensor for the analysis of nitrite in food was developed based on hemoglobin (Hb) covalently immobilized on the succinimide functionalized poly(n-butyl acrylate)-graphene [poly(nBA)-rGO] composite film deposited on a carbon-paste screen-printed electrode (SPE). The immobilized Hb on the poly(nBA)-rGO conducting matrix exhibited electrocatalytic ability for the reduction of nitrite with significant enhancement in the reduction peak at −0.6 V versus Ag/AgCl reference electrode. Thus, direct determination of nitrite can be achieved by monitoring the cathodic peak current signal of the proposed polyacrylic-graphene hybrid film-based voltammetric nitrite biosensor. The nitrite biosensor exhibited a reproducible dynamic linear response range from 0.05–5 mg L^−1^ nitrite and a detection limit of 0.03 mg L^−1^. No significant interference was observed by potential interfering ions such as Ca^2+^, Na^+^, K^+^, NH_4_^+^, Mg^2+^, and NO_3_^−^ ions. Analysis of nitrite in both raw and processed edible bird’s nest (EBN) samples demonstrated recovery of close to 100%. The covalent immobilization of Hb on poly(nBA)-rGO composite film has improved the performance of the electrochemical nitrite biosensor in terms of broader detection range, lower detection limit, and prolonged biosensor stability.

## 1. Introduction

Nitrite may occur naturally in waters or foods such as vegetables due to the use of nitrogen-based fertilizer, or may be added to foods to prevent the growth of foodborne pathogenic microorganisms, e.g., the Clostridium botulinum bacterium. Moreover, nitrite is also used extensively in processed food for a specific purpose, particularly to restore color or to improve flavor to a food. It has been proven that excessive nitrite levels in the blood can lead to oxidation of hemoglobin (Hb) [1,2]. Nitrite in the human body can also react with amines to form carcinogenic *N*-nitrosamines [3,4]. Edible bird’s nests (EBNs) containing elevated levels of harmful nitrite have been reported where swiftlet nests were found to contain more than 1000 mg L^−1^ of nitrite [5]. The continued intake of nitrite can be harmful to human health [6,7], particularly for Chinese pregnant mothers who eat them regularly during their pregnancy stage. As a precaution, a nitrite analysis for all types of EBNs, including “cave” nests and “house” nests, is necessary before they are exported to international markets.

In general, Hb consists of four polypeptide chains and each globin peptide chain contains an electroactive iron containing heme group in the Fe^2+^ state. Although Hb does not act as an electron carrier, its heme proteins are mainly useful because of their redox activity. It is well known that nitrite can irreversibly oxidize hemoglobin (HbFe^2+^) to form the methemoglobin (HbFe^3+^), thus making direct determination of nitrite possible by using Hb as the reagent [8]. This approach has been proven successful by using biosensors based on Hb immobilized on the titania sol-gel [9], gold colloid [10], chitosan [11], silica sol-gel [12], etc. In addition, Hb is commercially available and possesses a well-documented structure [9,13]. Besides, methemoglobin conjugated to poly(acylic acid) (PAA) in organic solvent [14] and being wrapped in PAA nanogel [15] has been investigated for its peroxidase activity for electrochemial applications. However, the shortcoming of Hb of not being able to transfer electron directly to the electrode surface is due to the fact that Fe^III^/Fe^II^ redox couple of the heme group is embedded deeply in the globin molecules, i.e., the protein subunits [10,16]. Accordingly, Hb requires a promoter or mediator in order to drive a direct electrochemical reaction [17]. Therefore, it is important to consider the type of electrode material or immobilization method that is suitable for Hb immobilization to allow electron transfer reaction to occur at the surface of the working electrode with a remarkable ability to preserve the bioactivity of the immobilized Hb molecules.

On the other hand, graphene based nanomaterials have shown interesting applications in electrochemical biosensors because it provides a promising platform for biomolecular detection. Due to its superb electron transfer properties and excellent electrochemical catalytic activity, the detection response of analytes incorporating graphene in the electrochemical reaction can be augmented to a higher level. Recent studies have shown that graphene can enhance direct electron transport between the redox center of hemoglobin and electrode surface [18]. This makes it possible for graphene to act as “electronic wires” between protein and the electrode surface without the need of any intermediate reagents [2]. Besides, its exceptional electrocatalytic activity could also help to increase the electrochemical performance of the graphene biosensor.

The common methods of Hb immobilization are by entrapment [7], electrostatic interaction [19], physical adsorption [8] and forming composite [20] with graphene. In this work we take a completely different approach, i.e., by covalently attaching Hb onto polyacrylic-graphene composite film for electrochemical quantitation of nitrite by differential pulse voltammetry (DPV) method. The Hb molecules were chemically grafted at the surface of poly(n-butyl acrylate) film that contained graphene as an effective method for improving the stability of the Hb. Thus, poly(n-butyl acrylate)-graphene [poly(nBA)-rGO] hybrid film was employed as the immobilization material for Hb. The poly(n-butyl acrylate) not only possesses a good film-forming ability but it can be modified to include succinimide moieties via photocuring on the membrane surface, which could then covalently bind to Hb molecules. This can enhance the sensitivity of the electrochemical nitrite biosensor. However, the polymer membrane alone could not provide good conductivity to the electrode, therefore the direct electron transfer of Hb reaction with nitrite to the SPE surface was achieved by introducing graphene nanosheets within the polyacrylic film. To prove the usefulness of the new biosensor for nitrite analysis, real EBN samples were used for recovery studies.

## 2. Materials and Methods

### 2.1. Chemicals

Aldrich supplied chemicals such as n-butyl acrylate (nBA), 2-2-dimethoxy-2-phenylacetophenone (DMPP), dimethylformamide (DMF) and 1,6-hexanediol diacrylate (HDDA). Sodium dihydrogen phosphate (NaH_2_PO_4_), sodium hydroxide (NaOH), acetic acid and acetyl acetone were purchased from Systerm. *N*-acryloxysuccinimide (NAS) and human hemoglobin (Hb) were obtained from Across and Sigma, respectively. Hydrazine monohydrate from Sigma-Aldrich (Zellwood, FL, USA) and disodium hydrogen phosphate (Na_2_HPO_4_) from Hamburg Chemical (Corporate Parkway, Center Valley, PA, USA). All the chemicals were of analytical grade and used without further purification. Standard buffer solutions were prepared with deionized water.

### 2.2. Instrumentation

The average thickness and morphology of the graphene nanosheets were examined with field emission scanning electron microscopy (FESEM, JEOL JSM-7800F (JEOL, Peabody, MA, USA)), whilst identification of functional group and chemical structure elucidation of grapene was done with Fourier-transform infrared spectroscopy (FTIR, Perkin Elmer Spectrum 400 (PerkinElmer, Waltham, MA, USA)). UV-Vis spectrophotometer, model Varian-Cary Win UV 50 was used to determine the nitrite concentration with Griess chemical reagent. CV (cyclic voltammetry) and DPV (differential pulse votammetry) experiments were performed with Autolab PGSTAT 12 potentiostat (Metrohm, Utrecht, The Netherlands). Carbon-paste screen-printed electrode (SPE) modified with poly(nBA)-rGO composite film was used as the working electrode. Platinum and Ag/AgCl electrodes were used as auxiliary and reference electrodes, respectively. All the potentials measured in this study were referred to Ag/AgCl electrode, and homogeneous solutions were prepared using Elma S30H sonicator bath.

### 2.3. Synthesis of Graphene Nanosheets

Graphene oxide (GO) was first prepared from graphite powders according to the modified Hummers and Offeman [21] and Kovtyukhova et al. [22] methods. The GO precursor was dispersed in water and added with an appropriate amount of KOH solution and some 0.25 mL of 100 mmol hydrazine monohydrate to produce a homogeneous suspension. The as-prepared GO suspension was later ultrasonicated until a clear solution was formed followed by heating in an oil bath overnight at 95 °C [23]. The as-synthesized reduced graphene oxide (rGO) or graphene nanosheets, which appeared as black solid precipitation was strained and washed sequentially with 500 mL of deionized water and 500 mL of acetone, and dried in a vacuum desiccator for 24 h. The physical and chemical properties of the rGO were then characterized based on FTIR and FESEM analyses.

### 2.4. Fabrication and Characterization of Electrochemical Nitrite Biosensor

**Figure 1:**
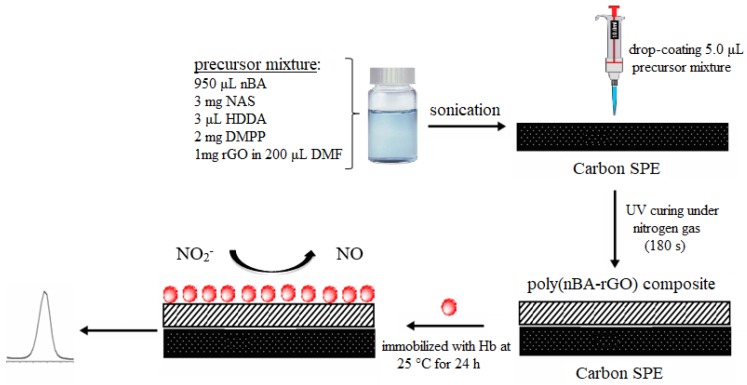
The schematic design and principle of operation of the electrochemical GM DNA biosensor based on poly(nBA-NAS)-rGO modified SPE.

A photocuring mixture of the polymer n-butyl acrylate-co-*N*-acryloxysuccinimide was prepared by mixing 950 μL nBA monomer, 3.0 μL HDDA crosslinking reagent, 2.0 mg DMPP and 3.0 mg NAS in a scintillation vial. About 1.0 mg of graphene in 200 μL DMF was then added into 125 μL of photocuring mixture solution and sonicated until a homogenous nBA-rGO mixture obtained. A total of 5.0 μL of the mixture was drop-coated onto the SPE and photocured under UV light and nitrogen gas atmosphere for 180 s to form the poly(nBA)-rGO composite film. The electron transfer kinetics on non-modified and modified carbon paste SPEs with rGO and polyacrylic matrix was investigated by CV using 0.1 M Fe(CN)_6_^3−/4−^ as the electrolyte. Hb solution was freshly prepared by dissolving 5 mg of Hb in 500 μL of 0.05 M potassium phosphate buffer at pH 7.0. The carbon paste SPE coated with the poly(nBA)-rGO hybrid film was then immersed in the Hb solution and left to react with the functionalized polymer film at room temperature (25 °C) for 24 h. Finally, the electrode was rinsed with abundant amount of deionized water to remove any unreacted Hb molecules from the electrode surface. The direct electrochemistry of the Hb immobilized on the acryloxysuccinimide functionalized acrylic-graphene composite film modified SPE was investigated by CV in 0.05 M potassium phosphate buffer (pH 7.0), and the potential application of the proposed Hb electrode for the detection of nitrite was explored with both CV and DPV methods. The schematic design of this Hb modified graphene nanosheets based electrode is shown in Figure 1.

### 2.5. Optimizing the Electrochemical Nitrite Biosensor Based on Poly(nBA)-rGO Supporting Matrix

The effect of composition ratio between rGO and photocuring mixture towards nitrite biosensor response was conducted by changing the volume ratio of rGO in DMF to photocuring mixture solution from 0.1:1 to 1:1. The CV measurement was carried out in 0.05 M potassium phosphate buffer at pH 5.5 containing 1 mg L^−1^ nitrite in the potential range of −1.0 V −0.2 V. The NAS loading effect was performed by varying the NAS loading in the photocuring mixture between 1 mg and 5 mg, whilst the Hb loading effect was done by alterating the amount of Hb from 2–25 mg in 500 μL of 0.05 M potassium phosphate buffer at pH 7.0 prior to immobilization procedure. The influence of pH on the direct electron transfer of immobilized Hb on the poly(nBA)-rGO-SPE was conducted using a series of 0.05 M potassium phosphate buffer from pH 5.0 to pH 7.0 as the measuring buffers towards the detection of 1 mg L^−1^ nitrite by DPV. Buffer capacity effect was performed by varying the potassium phosphate buffer (pH 5.5) concentration between 0.01 M and 0.1 M. The linear response range of the nitrite biosensor was then examined with a series of nitrite standard solutions with different concentrations from 0.01–10 mg L^−1^ nitrite in 0.05 M potassium phosphate buffer (pH 5.5). To investigate the effect of Hb immobilization time on the nitrite biosensor response, about 5 mg Hb was prepared in 500 μL of 0.05 M potassium phosphate buffer (pH 7.0), and the DPV response at −0.6 V was measured intermittently between 1 h and 24 h, at ambient temperature. The shelf life of the nitrite biosensor was studied by using electrodes stored at 4 °C. These electrodes were tested in triplicates with 1 mg L^−1^ of nitrite in 0.05 M potassium phosphate buffer (pH 5.5) at fixed intervals for up to a duration of 40 days where a substantial decline in the biosensor DPV response was observed at −0.6 V. To test the selectivity of the nitrite biosensor towards nitrite, several interfering ions commonly found in food samples, especially in bird’s nest, for example NH_4_^+^, Na^+^, K^+^, Ca^2+^, NO_3_^−^ and Mg^2+^ ions in 0.05 M potassium phosphate buffer, pH 5.5 at different molar ratios (1:50, 1:100, 1:200 and 1:300) towards nitrite were used. Significant interference to the biosensor response occurred if the changes of the signal were ±5% of the DPV peak current at −0.6 V for the signal produced by 1.45 × 10^−5^ M (1 mg L^−1^) nitrite.

### 2.6. Validation and Recovery Studies

A standard procedure employing a non-enzymatic method based on a Griess reagent was adopted to validate with the developed electrochemical biosensor for nitrite assay. The Griess reagent was prepared by reacting *N*-(1-naphthyl)ethylenediamine with sulfanilic acid. The Griess reagent is a colorless dye that turns pinkish shade when reacting with nitrite. The calibration curve for nitrite was established using standard nitrite concentrations within the range of 1–100 μM (0.046–4.6 mg L^−1^) with Griess reagent. About 300 μL of standard nitrite solution was mixed with 100 µL of Griess reagent and 2.6 mL of deionized water, and left for 30 min for reaction at 25 °C before the absorbance was measured at 548 nm by UV-Vis spectrophotometer. A blank reagent was prepared by mixing 100 μL of Griess reagent with 2.9 mL of deionized water.

Both processed and unprocessed edible bird’s nest (EBN) samples were used as food samples in validating the biosensor performance with the standard Griess method in quantitative assessment of nitrite content in foods. Unprocessed EBN was collected from local birdhouse, whilst the processed EBN was purchased from local hypermarket. Both processed and unprocessed EBNs were soaked in deionized water for 3 h at room temperature, and were strained through Whatman filter paper grade No. 1. The filtrates of the respective raw and processed EBNs were then spiked with standard nitrite from 0.3–2.0 mg L^−1^ and measured with the proposed biosensor at the potential of −0.6 V and Griess reagent system. The recovery of the spiked nitrite concentration by the nitrite biosensor was evaluated by calculating the percentage of recovery using Equation (1):

% Recovery = Cs/C × 100%(1)

where Cs is the concentration of nitrite determined by the biosensor and C is the actual concentration of nitrite spiked into the food samples.

## 3. Results and Discussion

### 3.1. Physical and Chemical Characterizations of the Synthesized Graphene

**Figure 2:**
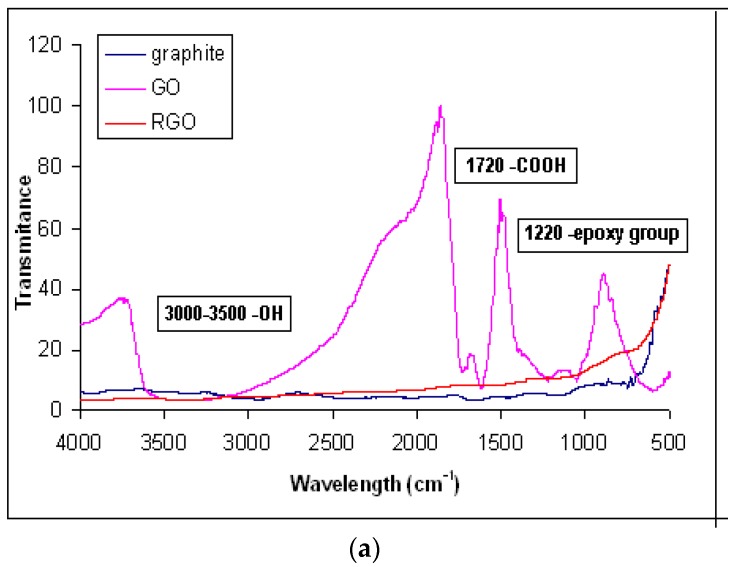

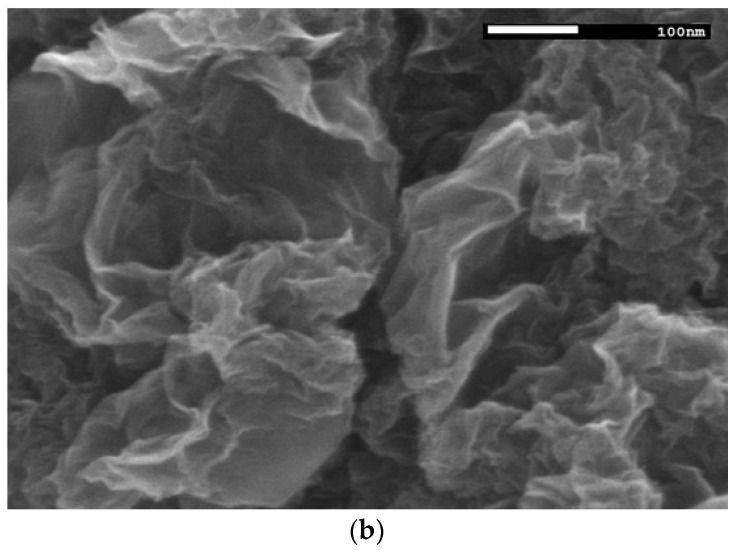
Fourier-transform infrared spectroscopy (FTIR) spectra of graphite, GO and rGO recorded by FTIR spectrometer (**a**) and field emission scanning electron microscopy (FESEM) image of rGO captured at an acceleration voltage of 3.0 kV and 150 k × magnification (**b**).

The as-synthesized rGO has been previously applied in the DNA biosensor fabrication and characterized by Raman spectroscopy to study the disorder and defects in both GO and rGO, and the results indicate the removal of oxygen functional groups in GO has created more defect sites in rGO [24]. Figure 2a shows the FTIR spectra of GO and rGO, and compared with that of the control sample by using graphite. A broad absorption band at 3430 cm^−1^ reveals O-H stretching vibrations, which indicates the hydroxyl functional group of GO. The FTIR absorption peak at 1720 cm^−1^ shows C=O stretching vibration, which implies the presence of oxygen-containing groups in GO. The epoxy functional group (C–O–C) of GO is indicated by the C–O (epoxy) and C–O (alkoxy) stretching vibration bands at 1220 cm^−1^ and 1051 cm^−1^, respectively. These functional groups were then reduced significantly after the reduction process, which suggests the successful reduction of GO to rGO. The as-synthesized rGO has a curvy and wrinkled graphene nanosheets at an average thickness of 2–4 nm, which signifies a complete exfoliation of graphite during ultrasonication treatment (Figure 2b).

### 3.2. Electrochemical Characteristics of Polyacrylic-Graphene Composite Film Coated Electrodes

When nitrite ion is present in an acidic medium, the immobilized Hb catalyzed the reduction of nitrite to form nitric oxide (NO), whilst hemoglobin oxidized to methemoglobin. As NO has a high affinity towards the iron heme complex, it then bound to the ferrous deoxyhemoglobin (HbFe^2+^) to form a ferrous nitrosyl complex, i.e., iron-nitrosyl-hemoglobin (HbFe^2+^-NO) [1,8]. The electrocatalytical reduction of nitrite on the Hb modified polyacrylic-graphene composite electrode [Hb-poly(nBA)-rGO-SPE] can be described by Equations (2) and (3) as below.

NO_2_^−^ + HbFe^2+^ + H^+^ → NO + HbFe^3+^ + OH^−^ (2)

HbFe^2+^ + NO → HbFe^2+^-NO (iron-nitrosyl-hemoglobin) (3)

This redox reaction can be monitored at a voltammetric peak potential of −0.6 V and thus direct quantification of nitrite can be performed via current measurement.

**Figure 3:**
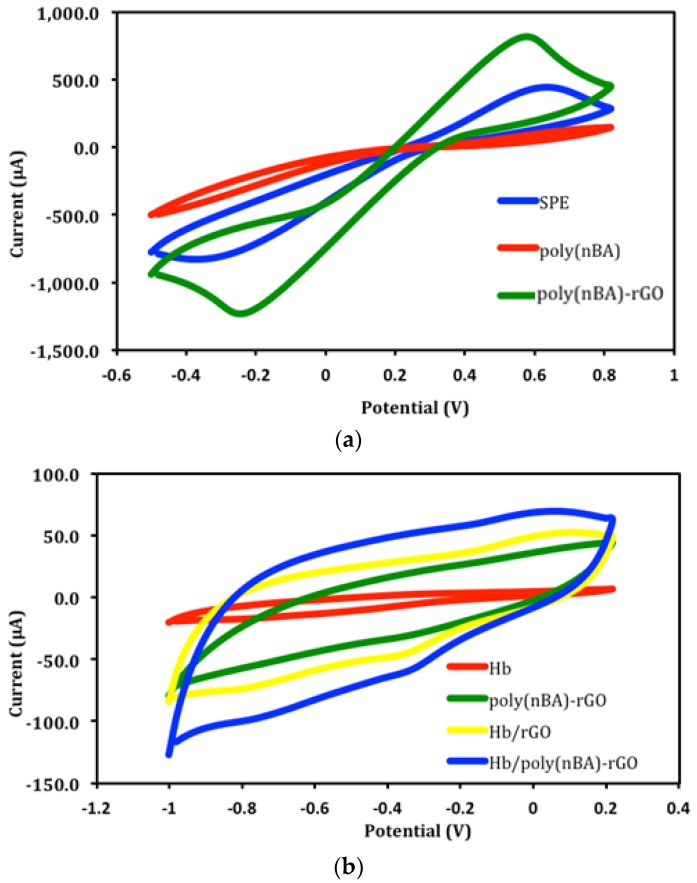

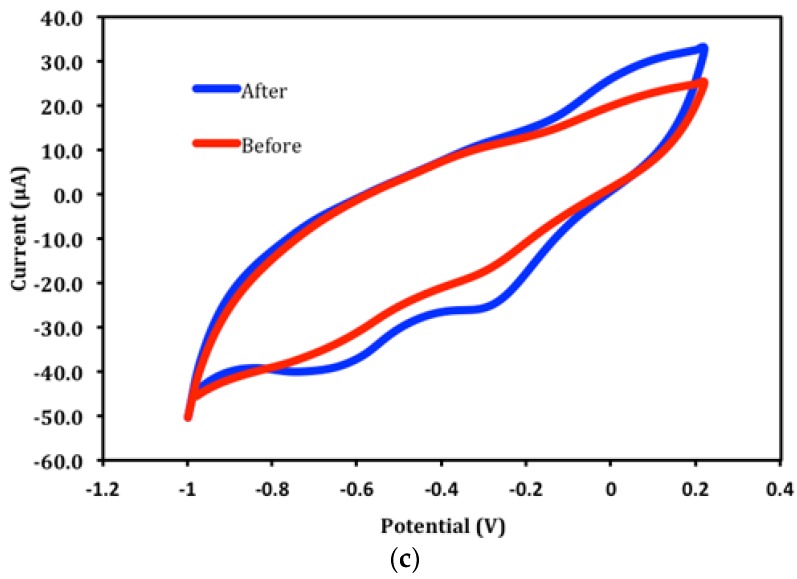
Cyclic voltammograms of bare screen-printed electrode (SPE), poly(nBA)-SPE and poly(nBA)-rGO-SPE in 0.1 M Fe(CN)_6_^3−/4−^ (**a**); CVs of Hb-SPE, poly(nBA)-rGO-SPE, Hb-rGO-SPE and b-poly(nBA)-rGO-SPE in 0.05 M PBS (pH 7.0) (**b**); CVs of Hb-poly(nBA)-rGO-SPE before and after reaction with 1 mg L^−1^ nitrite at pH 5.5 (**c**).

Cyclic voltammograms in Figure 3a show the detailed information about the electron transfer kinetics on non-modified and modified carbon paste SPEs with rGO and polyacrylic matrix in 0.1 M Fe(CN)_6_^3−/4−^ at a scan rate of 0.1 Vs^−1^. The rGO modified SPE demonstrated a considerable increase in the CV peak height and a decline in the peak-to-peak separation (ΔEp) compared to bare SPE. This suggests that the highly conductive graphene nanosheets have improved the electron transfer rate at the carbon paste SPE surface. Meanwhile, the slow electron transfer rate exhibited by the electrode with polyacrylic film alone indicates that the film is a non-conductive polymer [24]. However, a pair of well-defined redox peaks of Fe(CN)_6_^3−/4−^ couple was obtained when the electrode was coated with the poly(nBA)-rGO composite film.

The cyclic voltammogram of Hb-poly(nBA)-rGO-SPE in Figure 3b shows an inconspicuous peak at −0.3 V compared to poly(nBA)-rGO-SPE. This voltammetric peak was found located at the potential characteristic of the heme Fe^III^/Fe^II^ redox couple of the proteins [8]. This denotes the successful immobilization of Hb on the acrylic-graphene hybrid electrode. By comparing the CV of Hb-rGO-SPE with Hb-SPE, the presence of graphene has greatly augmented the electrochemical response of the embedded iron heme of Hb molecules immobilized on the SPE [2,8], and that the direct electron transfer was achieved for Hb immobilized on the rGO modified SPE. The addition of poly(nBA) with succinimide functional groups played an important role in increasing the number of Hb molecules immobilized on the electrode surface via an amide covalent bond, which can be formed between an amine functional group of Hb and a succinimide functional group of the acrylic polymer [17,25]. This was evidenced by the larger CV response of the Hb-poly(nBA)-rGO-SPE compared to Hb-rGO-SPE.

In acidic conditions, i.e., pH 5.5 using 0.05 M potassium phosphate buffer, the Hb-poly(nBA)-rGO-SPE exhibited a cathodic peak potential at −0.6 V in the presence of 1 mg L^−1^ nitrite (Figure 3c), whilst the generation of nitric oxide (NO) was thermodynamically favorable, and no CV peak was observed when the reaction medium was changed to neutral or basic conditions. This was attributed to the deficiency of proton as the main reason for the failure to generate NO at pH 7 and above. Hence, the CV results confirmed the cathodic peak potential at −0.6 V was ascribed to the reduction of nitrite to NO catalyzed by the immobilized Hb in acidic medium, whilst the characteristic peak potential of Hb heme group remained intact at −0.3 V. Similar electrochemical findings were observed by Liu et al. [7] at diffrerent pHs using a hydrogel supporting matrix for Hb immobilization on the glassy carbon electrode. Since oxygen is a major electroactive compound in natural oxygenated waters and can thus interfere with the developed biosensor in the measurement of nitrite in aqueous-based electrochemical system, and that oxygen can be removed by purging with an inert gas such as nitrogen or argon to avoid interference from oxygen reduction, reaction normally occurs at –0.1 V versus Ag/AgCl electrode.

### 3.3. Effects of rGO, NAS, and Hb Loadings on the Nitrite Biosensor Response

**Figure 4:**
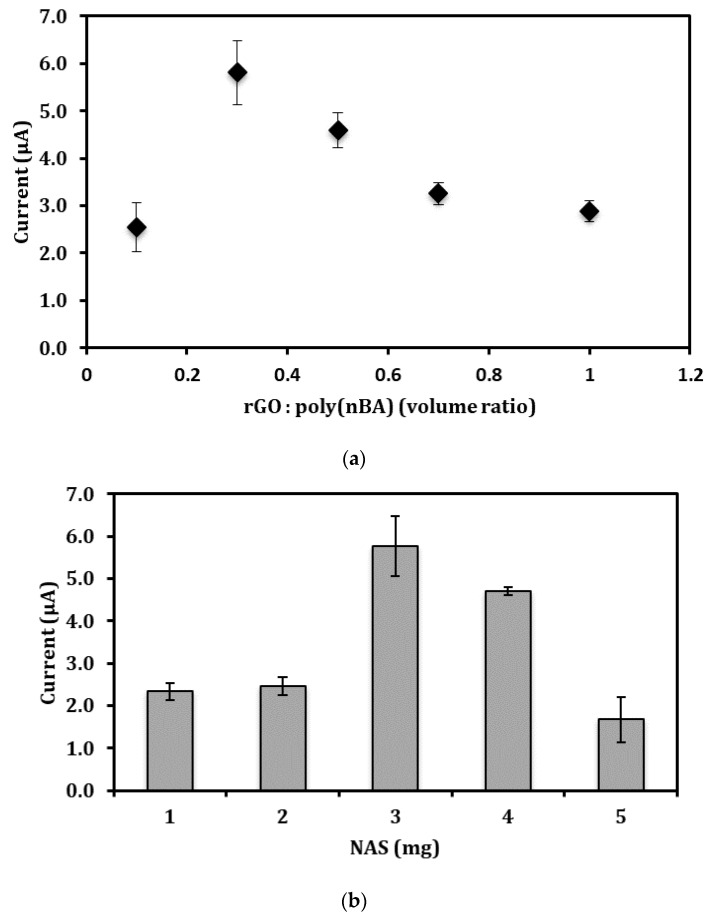

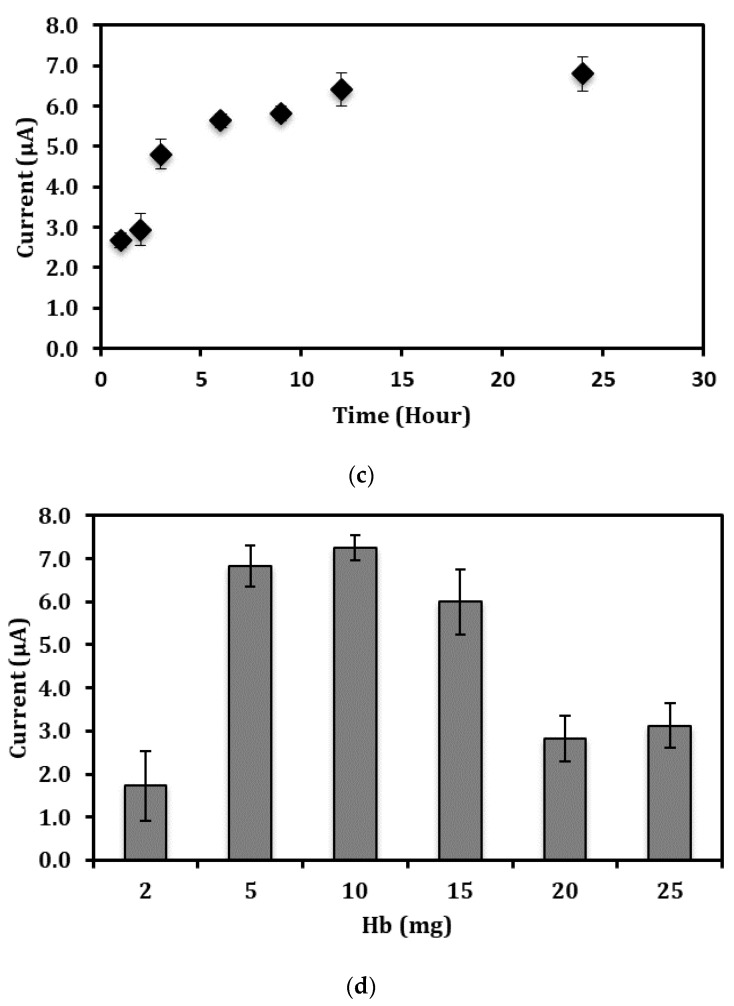
The composition ratio between rGO and acrylic precursor [rGO:poly(nBA)] (**a**), NAS (**b**) Hb immobilization time (**c**) and Hb concentrations’ effects (**d**) on the voltammetric nitrite biosensor response. The reaction was performed in the 0.05 M potassium phosphate buffer at pH 5.5 containing 1 ppm nitrite.

The beneficial properties of n-butyl acrylate (nBA), such as insoluble in water, plasticizer-free, photcurable and low glass transition temperature (Tg), thus provides a sticky characteristic to allow strong adhesion of the resulting acrylic polymer to the electrode surface [26,27,28,29]. In view of the insulating characteristic of the polyacrylate, the graphene-polymer composition must be optimized to allow optimum amplification of the electrochemical signal by the embedded graphene. As can be seen in Figure 4a, the reduction peak current of the nitrite biosensor at −0.6 V increased when the composition ratio between rGO in DMF and acrylic precursor [rGO:poly(nBA)] increased from 0.1 to 0.3 as more conductive pathways were generated by the increasing amount of graphene nanosheets loaded in the acrylic film, thus shortening the electron transfer distance and enhancing the electrical communications between redox centers of heme proteins and the electrode surface. However, the biosensor response progressively diminished as the rGO loading increased from a rGO:poly(nBA) volume ratio of 0.3 to 1.0 due to the overlapping layers of rGO, which has caused the blockage of electron transfer on the SPE surface. Therefore, the rGO:poly(nBA) volume ratio was maintained constant at 0.3 in the following optimization studies.

*N*-acryloxysuccinimide (NAS) possesses a succinimide functional group that can covalently bind with an amine group to form a peptide bond (–CO–NH–) [30,31]. Additionally, the NAS also has a C=C functional group, which allows it to form a copolymer with nBA. In view of the Hbs, which are protein molecules made from long chains of amino acid residues, and these amino acids containing amine (–NH_2_) and carboxyl (–COOH) functional groups, along with a side-chain (R group) specific to each amino acid. This allows spontaneous chemical adsorption of amine (–NH_2_) functional groups of Hb with succinimide functional group of poly(nBA)-rGO composite film modified SPE. Covalent immobilization of protein molecules, such as enzymes, has been shown in our previously reported study based on an optical enzyme-based urea biosensor using succinimide-modified acrylic microspheres as the supporting matrix [26]. The FTIR characterization result demonstrated characteristic IR absorption peaks of specific amide covalent bonds, which indicates the urease can be immobilized onto the surface of the acrylic microspheres by covalent binding because the NAS succinimide ester groups are highly reactive towards enzyme amino groups. The quantity of Hb molecules immobilized on the NAS-modified acrylic-rGO composite film is depending on the amount of NAS present in the composite film. Figure 4b shows the NAS loading profile of the nitrite biosensor. The cathodic peak current of the electrochemical nitrite biosensor increased with the increasing NAS loading from 1.0 mg to 3.0 mg. The increase in the amount of NAS led to the available of succinimide functional groups on the composite film for binding with Hb molecules. The biosensor response then declined when the NAS monomer was used in excess amount at 3 mg and above. This was due to the polymerization reaction that occurred between NAS monomers via a Michael addition reaction that involved nucleophilic enolates (succinimide group) and the ketone group conjugated alkenes electrophilic molecules, which exist at the end of the NAS molecule [32]. This reaction resulted in less succinimide functional groups that were available to serve as the active sites, thereby reducing the quantity of immobilized Hb at the poly(nBA)-rGO composite film surface [33,34,35,36].

Figure 4c shows the experimental results of the Hb immobilization duration on the acryloxysuccinimide functionalized acrylic-graphene composite film modified SPE. The DPV response of the nitrite biosensor gradually increased at the beginning of the experiment when the Hb immobilization duration was increased from 1–4 h. This was acsribed to the increasing number of Hb molecules covalently attached on the poly(nBA)-rGO-SPE to allow a greater electrocatalytical reduction rate of nitrite to occur at the electrode surface, and that the increment in the electrochemical signal with time can be perceived. The electrochemical biosensor reached a steady state response when the Hb immobilization duration was proceeded from 6–24 h as the available succinimide functional groups on the poly(nBA)-rGO composite film have almost entirely coupled to the Hb molecules. Therefore, no obvious signal change in the electrochemical catalytic response was observed after 6 h of Hb immobilization duration.

The amount of immobilized Hb on the surface of the poly(nBA)-rGO film relates to the sensitivity as well as the limit of detection of the nitrite biosensor. By increasing the amount of Hb immobilized on the poly(nBA)-rGO-SPE, it boosted up the catalytic reaction rate of the immobilized Hb, which was indicated by the increasing electrocatalytic nitrite reduction rate on the Hb-poly(nBA)-rGO-SPE, whereby the cathodic peak current increased between 2 mg and 10 mg Hb loadings (Figure 4d), after which the voltammetric peak signal dramatically declined with the further increase in Hb loading up to 25 mg. This was ascribed to the overloading of immobilized Hb on the electrode, and hindered the direct electrochemical reaction at the SPE surface. As Figure 4d indicates, 10 mg immobilized Hb gives the maximum voltammetric peak response, therefore 10 mg of Hb loading at the poly(nBA)-rGO-SPE was maintained in the next optimization studies of the electrochemical nitrite biosensor. The optimized Hb loading at 10 mg (rate constant, *k*_s_ = 50.22 s^−1^) was found to be far lower than the amount of immobilized Hb required by the study carried out by Liu et al. [8] using chitosan and dimethylformanide hydrogel as the immobilization matrix for an entrapment of 20 mg Hb (*k*_s_ = 58.77 s^−1^) on the glassy carbon electrode. This indicates only a very small amount of Hb is required for the analysis of nitrite with the proposed nitrite biosensor since the Hb molecules are immobilized at the surface of the electrode, and that they can have direct access to their analyte, hence tend to be much more sensitive compared to the thick membrane entrapment method that possesses more cross-link structures within the polymer matrix, which could prevent or slow the substrates from penetrating the membrane to react of the entrapped Hb.

### 3.4. Influence of pH and Buffer Concentration on the Direct Electrochemistry of Immobilized Hb

**Figure 5:**
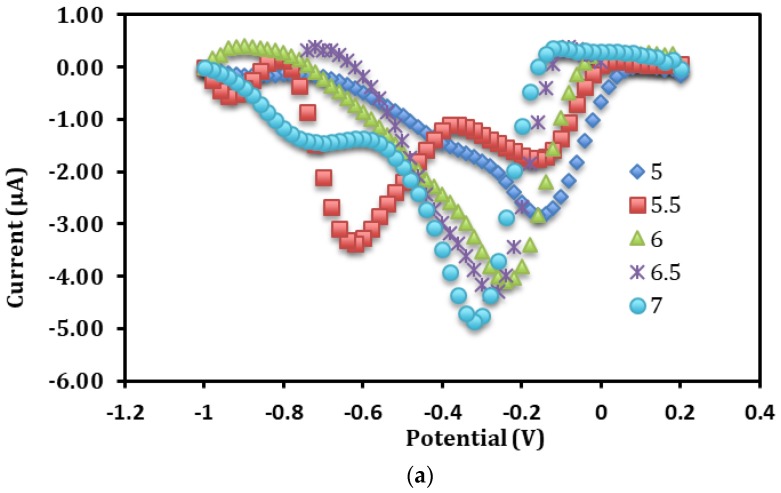

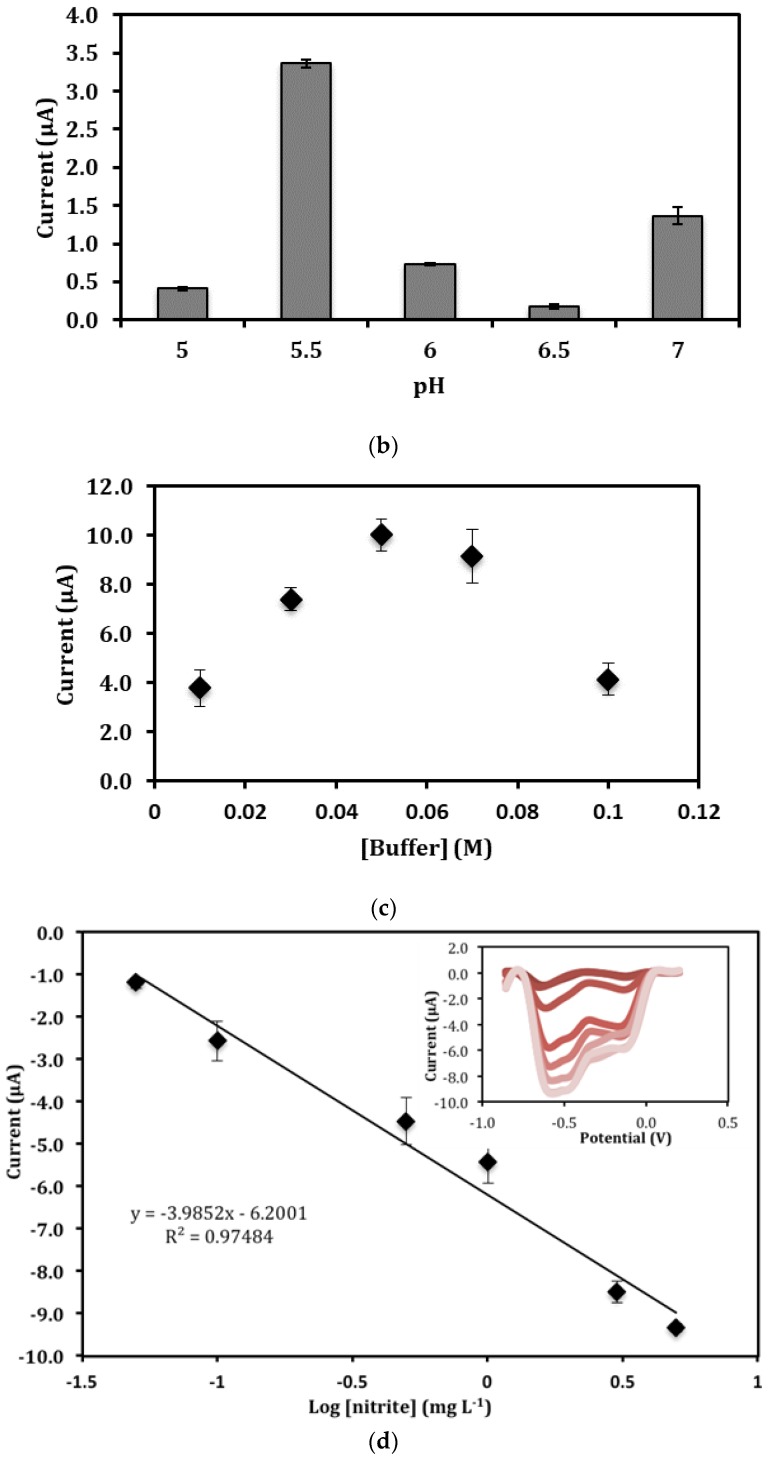
The differential pulse voltammograms of Hb-poly(nBA)-rGO-SPE in 0.05 M potassium phosphate buffer at different pHs (**a**). The DPV response of the Hb SPE in 0.05 m potassium phosphate buffer of different pHs (**b**) and buffer concentrations (**c**) containing 1 ppm nitrite at −0.6 V versus Ag/AgCl reference electrode. Calibration curve of the biosensor in the nitrite concentration range of 0.05–5 mg L^−1^ (*n* = 3), the inset shows the DPV signals of the Hb-poly(nBA)-rGO-SPE from 0.01–10 mg L^−1^ nitrite in 0.05 M potassium phosphate buffer at pH 5.5 (**d**).

The redox behavior of proteins is often strongly dependent on the pH of the surrounding solution [11,13]. As Figure 5a indicates, the characteristic DPV peak potential of the Fe^III^/Fe^II^ redox couple of Hb was noticed to shift to more negative potentials with the rise in pH from pH 5.0 to pH 7.0, which correspond to the electron transfer reaction accompanied by the exchange of proton [7]. A reduction peak was perceived at about −0.6 V when the reaction medium was adjusted to pH 5.5, indicating that the immobilized Hb catalyzed the direct reduction of 1 mg L^−1^ nitrite at the electrode surface. The reduction peak at −0.6 V in 0.05 M potassium phosphate buffer electrolyte buffer (pH 5.5) was found to be the most dominant DPV signal for the direct electrochemistry of immobilized Hb on the poly(nBA)-rGO-SPE compared to other pH conditions (Figure 5b), hence pH 5.5 was used to quantify the concentration of nitrite in the subsequent experiments by DPV method.

The ionic strength of the solution can affect the solubility of the protein molecules. When the potassium phosphate buffer concentration (pH 5.5) was increased from 0.01–0.05 M, the increasing ionic strength of the potassium phosphate buffer solution promoted the of Hb molecules (Figure 5c). This allowed the immobilized Hb to spontaneously resume its three-dimensional structure, thus regaining the function of the catalytically active group reactivity as its native form, and resulting accessibility of the immobilized Hb electroactive centers. Nevertheless, high salt concentration i.e., between 0.07 M and 0.1 M ionic strength resulted in a reduction in the solubility of proteins, leading to the clot formation of Hb [36]. Consequently, the high ionic strength buffer affected the activity and structural stability of the immobilized Hb. Therefore, a 0.05 M potassium phosphate buffer at pH 7.0 was utilized as the optimum phosphate buffer concentration in all nitrite biosensor optimization studies.

The electrocatalytic reduction of nitrite by Hb immobilized poly(nBA)-rGO-SPE is exemplified in Figure 5d. The DPV peak current of the Hb SPE at −0.6 V increased consistently with the increasing of the nitrite concentration from 0.01–10 mg L^−1^, and the dynamic linear response range of the nitrite biosensor was determined between 0.05 mg L^−1^ and 5 mg L^−1^ (*R*^2^ = 0.97) with a limit of detection (LOD) of 0.03 mg L^−1^ nitrite. The LOD was calculated based on the concentration equivalent to the DPV signal of blank plus three times standard deviation of the blank. The reproducibility relative standard deviation (RSD) value for each calibration point was calculated in the range of 7–9% (*n* = 7).

### 3.5. Long Term Stability of the Biosensor Response and Interference Studies

**Figure 6:**
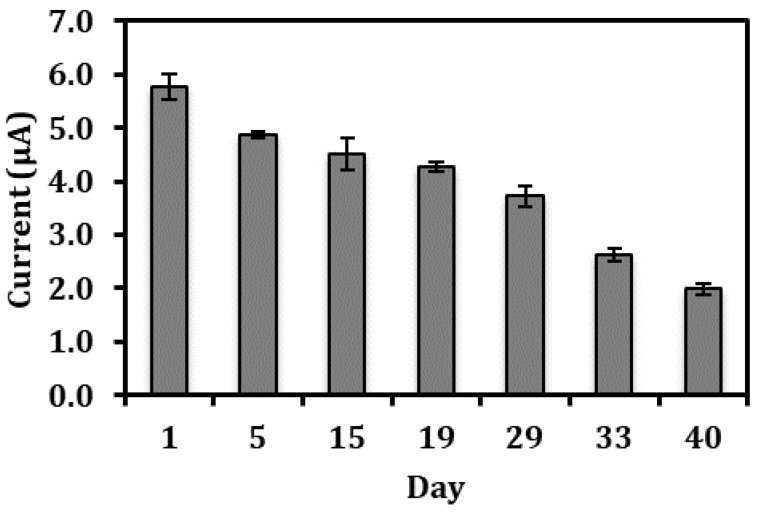
The shelf life profile of the electrochemical nitrite biosensor over a period of 40 days.

The lifetime of the electrochemical nitrite biosensor is presented in Figure 6. The nitrite biosensor based on Hb-poly(nBA)-rGO-SPE was able to remain 95% of its initial value for 1 mg L^−1^ nitrite detection in 0.05 M potassium phosphate buffer (pH 5.5) after one week of storage period at 4 °C. Two weeks later, the biosensor still retained about 85% of its original response. Even after a storage of up to 40 days, the biosensor response has only declined to approximately 50% of its initial value. The slow loss of response may be attributed to the denaturation of the proteins upon prolonged storage. The satisfactory long lifetime of the biosensor was ascribed to the use of NAS modified polyacrylic film that has permitted covalent attachment between succinimide from the polymer and amine groups of the Hb, thus providing physical and chemical protections to the immobilized Hb and reducing the biomolecule leaching problem whilst maintaining the bioelectrocatalytic activity of the immobilized Hb [26].

Table 1 shows the effect of interfering ions on the nitrite biosensor response at various molar ratios between nitrite and interferents. The study showed that the interference effect of those common cations that normally co-exist with nitrite in food was not significant (<±5%) up to a molar ratio of 1:200 for most cations. K^+^ and Na^+^ ions showed a significant interference effect to the detection of nitrite at the molar ratio of 1:100 and 1:200 as the presence of a high concentration of these cations has increased the ionic strength of the reaction medium, and that it reduced the electrochemical catalytic activity of the immobilized Hb for the reduction nitrite [37].

### 3.6. Validation of the Electrochemical Biosensor for the Detection of Nitrite in Food Samples

The feasibility of the proposed electrochemical biosensor based on Hb immobilized acrylic-graphene composite film on the carbon SPE for direct detection of nitrite concentration in food samples was evaluated with a UV-Vis spectrophotometric method using the Griess reagent. Aqueous extracts of processed and unprocessed bird’s nests samples were spiked with different concentrations of nitrite within the calibration range of the biosensor and later assessed with both biosensor and Griess methods. A comparison of these two methods for estimating the nitrite concentrations in EBN samples demonstrated good correlations between the biosensor and Griess methods. For the processed EBN samples, the correlation was y = 0.8667x + 0.108 (*R*^2^ = 0.9959) and for the unprocessed samples, y = 0.9156x + 0.0565 (*R*^2^ = 0.9987). Thus, the nitrite biosensor analytical performance for nitrite determination is comparable with the standard Griess method regardless of the presence of significant impurities in the EBN samples. Furthermore, the recovery of spiked nitrite in the EBN samples by the electrochemical biosensor showed recovery percentages of 100–109% (Table 2), suggesting there is a practical use of this biosensor for the accurate and reliable determination of nitrite in food samples. According to Rule 147 of Food Regulations 1985 in Malaysia, the permissible nitrite level in food such as meat products must be below 200 ppm, and the Malaysian Ministry of Health has advocated the nitrite limit in both drinking and natural waters at <10 ppm, whilst the permissible level of nitrite in drinking level is <0.1 ppm as per World Health Organization limit [5]. And, the dynamic linear response range of the developed electrochemical nitrite biosensor (i.e., 0.05–5 mg L^−1^, LOD = 0.03 mg L^−1^) covers the useful analytical range, which is feasible for practical analysis.

### 3.7. A Comparison of Analytical Performance of Nitrite Biosensor with Several Reported Electrochemical Nitrite Biosensors

Both electrochemical and optical based chemosensors or biosensors for nitrite have been reported before [5,38,39,40], but only previously reported HB-based electrochemical biosensors are presented here for comparison with the developed nitrite biosensor. The analytical performance of these electrochemical nitrite biosensors is shown in Table 3. The nitrite biosensor based on poly(nBA)-rGO composite film with Hb immobilized has allowed the determination of nitrite at a much lower concentration range, a better detection limit and improved long term stability when compared with many other reported electrochemical nitrite biosensors that involved the non-covalent immobilization of Hb in graphene matrices, e.g., graphene oxide/Au nanoparticles/multiwalled carbon nanotubes composite film [21], poly (*N*-isopropylacyamide-co-3-methacryloxypropyltrimethoxysilane) (PNM) film [7], graphene-AuNP composites [20], and graphene nanosheet with chitosan-N, *N*-Dimethylformamide (CS-DMF) matrix [19]. The exceptionally good analytical performance, particularly improvement in long term stability exhibited by the biosensor reported here, was mainly attributed to the succinimide-functionalized polyacrylic film that has allowed the covalent attachment of Hb and increased the stability and loading capacity of Hb on the electrode surface. Furthermore, excellent conductivity by the graphene that eased the direct electron transmission between the redox center of Hb to the surface of SPE also played a role in enhancing the sensitivity and stability of the biosensor.

## 4. Conclusions

Covalent immobilization of Hb is an important strategy to retain as much as possible of the Hb molecules on the electrode surface besides retaining their biochemical properties to achieve the direct determination of nitrite. This work has demonstrated that such a strategy has allowed a much improved performance of the nitrite biosensor based on polyacrylic-graphene hybrid sensing film. This biosensor has shown application for the analysis of nitrite in edible bird’s nest food samples. This can be a cost effective analytical method for such food items. In addition, the nitrite biosensor is disposable and makes them particularly suitable for high-throughput monitoring of nitrite contaminant in foods.

## Figures and Tables

**Table 1:** The effect of interfering ions on the nitrite biosensor DPV response at −0.6 V towards the detection of 1.45 × 10^−5^ M (1 mg L^−1^) nitrite in 0.05 M potassium phosphate buffer (pH 5.5).

Interfering Ions		Biosensor Response (%)	
	1:50	1:100	1:200
Na^+^	4.26 ± 0.07	8.78 ± 0.04	16.85 ± 0.25
Ca^2+^	3.17± 0.46	4.39 ± 0.25	7.23 ± 0.10
K^+^	4.81 ± 0.07	10.92 ± 0.51	16.74 ± 0.17
Mg^2+^	3.17 ± 0.46	3.88 ± 0.30	4.20 ± 0.77
NH_4_^−^	1.31 ± 0.01	2.13 ± 0.26	3.17± 0.46
NO_3_^−^	1.68 ± 0.03	3.92 ± 0.32	4.68 ± 0.03

**Table 2:** The recovery of spiked nitrite in the processed and unprocessed edible bird’s nest (EBN) samples using the electrochemical nitrite biosensor (*n* = 3).

Spiked Nitrite (mg L^−1^)	Nitrite Concentration Determined by Biosensor (mg L^−1^)	Recovery (%)	Nitrite Concentration Determined by Biosensor (mg L^−1^)	Recovery (%)
	Processed EBN		Unprocessed EBN	
0.3	0.31	103	0.30	100
0.5	0.51	102	0.52	104
0.7	0.72	103	0.71	101
1.0	1.02	102	1.09	109
2.0	2.15	108	2.04	102

**Table 3:** A comparison of poly(nBA)-rGO/SPE based on electrochemical Hb biosensor with other previously reported studies on electrochemical Hb biosensor for nitrite determination.

Immobilization Matrix Materials	Linear Range (mM)	Limit of Detection (mM)	Long Term Stability (Day)	Reference
Poly(nBA)-rGO	0.07–0.7	0.04	14	This study
Graphene oxide/Au nanoparticles/multiwalled carbon nanotubes nanocomposite film	5.0–1700	0.2	14	[21]
Graphene-AuNP composites	0.05–1000	0.01	30	[19]
PNM modified glass carbon electrodes	0.11–1.88	0.1	30	[7]
Graphene nanosheet modified electrode by CS-DMF	0.3–55	0.18	-	[20]

## References

[1] Kim-Shapiro D.B., Gladwin M.T., Patel R.P., Hogg N. (2005). The reaction between nitrite and hemoglobin: The role of nitrite in hemoglobin-mediated hypoxic vasodilation. J. Inorg. Biochem..

[2] Yue R., Lu Q., Zhou Y. (2011). A novel nitrite biosensor based on single-layer graphene nanoplatelet-protein composite film. Biosens. Bioelectron..

[3] Hong J., Dai Z. (2009). Amperometric biosensor for hydrogen peroxide and nitrite based on hemoglobin immobilized on one-dimensional gold nanoparticle. Sens. Actuators B Chem..

[4] Mani V., Periasamy A.P., Chen S.M. (2012). Highly selective amperometric nitrite sensor based on chemically reduced graphene oxide modified electrode. Electrochem. Commun..

[5] Nur Syarmim M.N., Tan L.L., Lee Y.H., Chong K.F., Saiful Nizam T. (2016). Acrylic microspheres-based optosensor for visual detection of nitrite. Food Chem..

[6] Chen X., Wang F., Chen Z. (2008). An electropolymerized Nile Blue sensing film-based nitrite sensor and application in food analysis. Anal. Chim. Acta.

[7] Sun Y., Wang S. (2009). An amperometric nitrite biosensor based on the bioelectrocatalysis of hemoglobin incorporated in sol-gel film. Am. J. Biomed. Sci..

[8] Liu P., Zhang X.H., Feng L.J., Xiong H.Y., Wang S.F. (2011). Direct electrochemistry of hemoglobin on graphene nanosheet-based modified electrode and its electrocatalysis to nitrite. Am. J. Biomed. Sci..

[9] Yang W., Bai Y., Li Y., Sun C. (2005). Amperometric nitrite sensor based on hemoglobin/colloidal gold nanoparticles immobilized on a glassy carbon electrode by a titania sol-gel film. Anal. Bioanal. Chem..

[10] Gu H.Y., Yu A.M., Chen H.Y. (2001). Direct electron transfer and characterization of hemoglobin immobilized on a Au colloid–cysteamine-modified gold electrode. J. Electroanal. Chem..

[11] Huang H., Hu N., Zeng Y., Zhou G. (2002). Electrochemistry and electrocatalysis with heme proteins in chitosan biopolymer films. Anal. Biochem..

[12] Wang Q., Lu Q., Yang B. (2004). Direct electrochemistry and electrocatalysis of hemoglobin immobilized on carbon paste electrode by silica sol-gel film. Biosens. Bioelectron..

[13] Ma G.X., Lu T.H., Xia Y.Y. (2007). Direct electrochemistry and bioelectrocatalysis of hemoglobin immobilized on carbon black. Bioelectrochemistry.

[14] Zore O.V., Lenehan P.J., Kumar C.V., Kasi R.M. (2014). Efficient biocatalysis in organic media with hemoglobin and poly(acrylic acid) nanogels. Langmuir.

[15] Ghimire A., Zore O.V., Thilakarathne V.K., Briand V.A., Lenehan P.J., Lei Y., Kasi R.M., Kumar C.V. (2015). “Stable-on-the-table” biosensors: Hemoglobin-poly (acrylic acid) nanogel bioelectrodes with high thermal stability and enhanced electroactivity. Sensors.

[16] Liu H.H., Wan Y.Q., Zou G.L. (2006). Direct electrochemistry and electrochemical catalysis of immobilized hemoglobin in an ethanol–water mixture. Anal. Bioanal. Chem..

[17] Wen Y., Wu H., Chen S., Lu L., Shen H., Jia N. (2009). Direct electrochemistry and electrocatalysis of hemoglobin immobilized in poly(ethylene glycol) grafted multi-walled carbon nanotubes. Electrochim. Acta.

[18] Wen W., Chen W., Ren Q.Q., Hu X.Y., Xiong H.Y., Zhang X.H., Zhao Y.D. (2012). A highly sensitive nitric oxide biosensor based on hemoglobin–chitosan/graphene–hexadecyltrimethylammonium bromide nanomatrix. Sens. Actuators B Chem..

[19] Jiang J.J., Fan W.J., Du X.Z. (2014). Nitrite electrochemical biosensing based on coupled graphene and gold nanoparticles. Biosens. Bioelectron..

[20] Wang Y., Bi C.Y. (2014). A novel nitrite biosensor based on direct electron transfer of hemoglobin immobilized on a graphene oxide/Au nanoparticles/multiwalled carbon nanotubes nanocomposite film. RSC Adv..

[21] Hummers W.S., Offeman R.E. (1958). Preparation of Graphitic Oxide. J. Am. Chem. Soc..

[22] Kovtyukhova N.I., Ollivier P.J., Martin B.R., Mallouk T.E., Chizhik S.A., Buzaneva E.V., Gorchinskiy A.D. (1999). Layer-By-Layer Assembly of Ultrathin Composite Films from Micron-Sized Graphite Oxide Sheets and Polycations. Chem. Mater..

[23] Stankovich S., Piner R.D., Chen X.Q., Wu N.Q., Nguyen S.T., Ruoff R.S. (2006). Stable aqueous dispersions of graphitic nanoplatelets via the reduction of exfoliated graphite oxide in the presence of poly(sodium 4-styrenesulfonate). J. Mater. Chem..

[24] Raja Zaidatul A.R.J., Lee Y.H., Tan L.L., Chong K.F. (2018). A Biosensor for Genetic Modified Soybean DNA Determination via Adsorption of Anthraquinone-2-sulphonic Acid in Reduced Graphene Oxide. Electroanalysis.

[25] Ulianas A., Lee Y.H., Musa A., Lau H.Y., Zamri I., Tan L.L. (2014). A regenerable screen-printed DNA biosensor based on acrylic microsphere–gold nanoparticle composite for genetically modified soybean determination. Sens. Actuators B Chem..

[26] Ulianas A., Lee Y.H., Musa A. (2011). A biosensor for urea from succinimide-modified acrylic microspheres based on reflectance transduction. Sensors.

[27] Lee Y.H., Alva S., Musa A. (2004). Ammonium ion sensor based on photocured and self-plasticising acrylic films for the analysis of sewage. Sens. Actuators B Chem..

[28] Lee Y.H., Hall E. (2000). Methacrylic–acrylic polymers in ion-selective membranes: Achieving the right polymer recipe. Anal. Chim. Acta.

[29] Sharina A.H., Lee Y.H., Musa A. (2008). Effects of Gold Nanoparticles on the Response of Phenol Biosensor Containing Photocurable Membrane with Tyrosinase. Sensors.

[30] Ulianas A., Lee Y.H., Sharina A.H., Tan L.L. (2012). An electrochemical DNA microbiosensor based on succinimide-modified acrylic microspheres. Sensors.

[31] Chaix C., Pacard E., Elaïssari A., Hilaire J.-F., Pichot C. (2003). Surface functionalization of oil-in-water nanoemulsion with a reactive copolymer: Colloidal characterization and peptide immobilization. Colloids Surf. B Biointerfaces.

[32] Chen J., Chiu S. (2000). A poly(*N*-isopropylacrylamide-co-*N*-acryloxysuccinimide-co-2-hydroxyethyl methacrylate) composite hydrogel membrane for urease immobilization to enhance urea hydrolysis rate by temperature swing. Enzyme Microb. Technol..

[33] D’Agosto F., Charreyre M.T., Pichot C. (2001). Side-Product of *N*-Acryloyloxysuccinimide Synthesis or Useful New Bifunctional Monomer?. Macromol. Biosci..

[34] Yew P.L., Lee Y.H. (2010). A Potentiometric Formaldehyde Biosensor Based on Immobilization of Alcohol Oxidase on Acryloxysuccinimide-modified Acrylic Microspheres. Sensors.

[35] Liu X.Q., Guan Y.P., Liu H.Z., Ma Z.Y., Yang Y., Wu X.B. (2005). Preparation and characterization of magnetic polymer nanospheres with high protein binding capacity. J. Magn. Magn. Mater..

[36] Ngounou B., Neugebauer S., Frodl A., Reiter S., Schuhmann W. (2004). Combinatorial synthesis of a library of acrylic acid-based polymers and their evaluation as immobilisation matrix for amperometric biosensors. Electrochim. Acta.

[37] Ling T.L., Ahmad M., Heng L.Y. (2011). An amperometric biosensor based on alanine dehydrogenase for the determination of low level of ammonium ion in water. J. Sens..

[38] Turdean G.L., Szabo G. (2015). Nitrite detection in meat products samples by square-wave voltammetry at a new single walled carbon naonotubes–myoglobin modified electrode. Food Chem..

[39] Yildiz G., Nevin O., Ayca O., Filiz S. (2014). Voltammetric determination of nitrite in meat products using polyvinylimidazole modified carbon paste electrode. Food Chem..

[40] Ensafi A.A., Maryam A. (2012). Highly selective optical nitrite sensor for food analysis based on Lauth’s violet–triacetyl cellulose membrane film. Food Chem..

